# PfAlba1: master regulator of translation in the malaria parasite

**DOI:** 10.1186/s13059-015-0795-x

**Published:** 2015-10-08

**Authors:** Evelien M. Bunnik, Karine G. Le Roch

**Affiliations:** Department of Cell Biology and Neuroscience, Institute for Integrative Genome Biology, Center for Disease Vector Research, University of California Riverside, 900 University Avenue, Riverside, CA 92521 USA

## Abstract

During the asexual replication cycle of the malaria parasite *Plasmodium falciparum*, the RNA-binding protein PfAlba1 binds and stabilizes a subset of transcripts for translation at a later time point.

Please see related Research article: http://www.genomebiology.com/2015/16/1/212

## Post-transcriptional gene regulation in the malaria parasite

Every year, an estimated 198 million people contract malaria and 584,000 people die as a result of the disease. Treatment of malaria is still essential to reduce morbidity and mortality, but is compromised by the spread of drug-resistant malaria parasites. Given the pressing need for novel antimalarial drugs, the regulation of gene expression in the human malaria parasite, *Plasmodium falciparum*, has been the subject of considerable interest. A better understanding of gene regulation and the identification of important players in this process have the potential to reveal novel drug targets.

During the stage of the parasite life cycle responsible for symptomatic disease and mortality in humans, the parasite replicates inside red blood cells in 48-hour cycles. After invasion of a red blood cell, high levels of transcription and translation are needed to generate the building blocks for approximately 16 daughter parasites. Because the *Plasmodium* genome encodes very few transcription factors, it has long been a mystery how the parasite regulates the cascade of gene expression observed in transcriptomics analyses. Recently, the focus has shifted towards post-transcriptional mechanisms of gene regulation. Several independent studies have shown that a subset of genes is not immediately translated following transcription [1–4], indicating that post-transcriptional regulation indeed plays an important role in timing the moment of translation for these transcripts. Although a handful of RNA-binding proteins involved in post-transcriptional regulation during the blood stage have been identified [5, 6], their function remains largely unknown. For other stages of the parasite life cycle, the process of translational repression and the role of RNA-binding proteins are better understood, for example in gametocytes that differentiate from asexual blood-stage parasites. In female gametocytes, hundreds of transcripts are stored and translationally repressed in ribonucleoprotein complexes until the parasite has sexually reproduced and matured into an ookinete in the mosquito midgut [7]. Several RNA-binding proteins that regulate this process have been identified, of which DOZI and CITH seem particularly important [8].

In a new study published in *Genome Biology*, Vembar and colleagues [9] present PfAlba1 as a key regulator of translation during the blood stage of the human malaria parasite life cycle. Their data support a model in which this protein binds and stabilizes at least 100 transcripts — and possibly many more — and releases them for translation at a later time point. A substantial proportion of these transcripts encode invasion genes that are needed at the late stage of the cell cycle to prepare for invasion of a new red blood cell. As PfAlba1 has not yet been implicated in translational repression during other parasite stages, it may thus be a specific post-transcriptional regulator of the asexual blood stage.

## Fishing for transcripts regulated by PfAlba1

Earlier work by Vembar and colleagues [10] revealed that the four members of the PfAlba protein family bind to RNA in vitro and that PfAlba1, 2, and 4 are located in distinct foci in the cytoplasm. Such foci could represent ribonucleoprotein complexes involved in mRNA storage or processing.

**Fig. 1:**
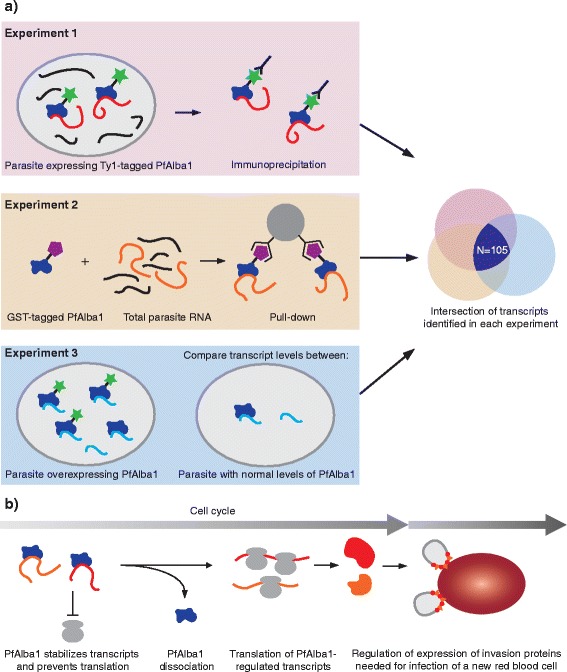
Post-transcriptional regulation by PfAlba1 in the human malaria parasite. **a** Three complementary strategies to determine which transcripts are post-transcriptionally regulated by PfAlba1 (see text for details). Transcripts identified in all three experiments are considered highly confident targets of PfAlba1. **b** Model describing the function of PfAlba1. Transcripts bound by PfAlba1 are stabilized and protected from translation. At a later time point in the cell cycle, transcripts are released and translated. A relatively large fraction of genes under post-transcriptional regulation by PfAlba1 encode invasion proteins that are needed at the late stage of the cell cycle to prepare the parasite for entry of a new host cell

The current study shows that removal of the gene encoding PfAlba1 or knockdown of PfAlba1 protein is incompatible with parasite viability, indicating that this protein is essential for parasite survival. To decipher the role of PfAlba1 in mRNA homeostasis, the authors performed a series of three complementary experiments aimed at identifying the transcripts that are bound by PfAlba1 (Fig. 1a). In the first experiment, a tagged version of PfAlba1 was expressed in the parasite and immunoprecipitated together with its bound mRNA, resulting in the identification of 1665 target transcripts. In the second experiment, recombinant PfAlba1 protein with a different tag was expressed in a bacterial system, purified, and then allowed to bind to total parasite RNA. Under these conditions, 1927 transcripts associated with PfAlba1. Finally, in the third experiment, PfAlba1 was expressed at higher than normal levels in the parasite and the effect of this overexpression on transcript abundance was measured. A total of 926 transcripts showed differential abundance in the presence of elevated levels of PfAlba1.

The strength of this study comes from combining these three different approaches to evaluate the function of PfAlba1. Each experiment will detect a number of false positives — transcripts that may nonspecifically interact with PfAlba1 under certain experimental conditions but that are not bona fide targets of this RNA-binding protein. However, transcripts that are detected in all three experiments are highly likely to be true targets regulated by PfAlba1. At the intersection of the three experiments were 105 transcripts, a relatively large fraction of which encodes components of the invasion machinery.

## Catch and release

To validate the role of PfAlba1 in regulating invasion gene transcripts, the authors then performed a combination of mRNA and protein detection experiments at two different time points during the blood-stage cell cycle. At the earlier time point, invasion transcripts were bound by PfAlba1 and the corresponding proteins were not detected. At the later time point, the transcripts were no longer associated with PfAlba1, while the protein products were detectable. These results show that the binding of PfAlba1 to these mRNA products stabilizes the transcripts and prevents translation (Fig. 1b). When the time is right, the transcripts are released and translated.

## A sea of unknowns

The 105 highly confident targets of PfAlba1 are likely to represent only a fraction of all transcripts that are regulated by this protein. A comparison of PfAlba1-associated transcripts detected here with other datasets of post-transcriptionally regulated genes [2, 3] showed a substantial overlap, indicating that PfAlba1 is an important player in the field of RNA homeostasis. However, PfAlba1 is probably not a lone star, but is likely to be part of a team of RNA-binding proteins that collectively regulate translational repression*.* For example, CAF1 of the CCR4–Not complex, a key mediator of mRNA decay in eukaryotes, has been shown to regulate transcript abundance for approximately 20 % of the parasite genome, including many proteins involved in egress and invasion [5]. PfAlba proteins are known to form dimers among themselves and may be part of larger complexes of RNA-binding proteins that could contain post-transcriptional regulators, such as DOZI and CITH, that are important in other stages of the parasite’s life cycle. PfAlba1 may thus contribute to mRNA stabilization by direct binding to transcripts, or by indirect association within large ribonucleoprotein complexes.

Another important aspect that remains to be elucidated is how the release of transcripts from PfAlba1 is regulated. Post-translational modifications of PfAlba1, such as phosphorylation or acetylation, may change its binding capacity and result in the dissociation from mRNA. Identifying the enzymes involved in this process would enable us to put together an important part of the gene regulation puzzle in *P. falciparum*.

## Outlook

The dependency of *P. falciparum* on PfAlba1 for its survival makes this protein an excellent target for novel antimalarial drugs. Such drugs could either target PfAlba1 directly, or block the release of transcripts from PfAlba1 during the late stage of the cell cycle, presumably mediated by post-translational modification of PfAlba1. This latter scenario would severely disrupt the expression of invasion genes necessary for infection of new red blood cells and thus break the replication cycle. Finally, it will be important to understand how the expression of genes that are currently investigated as vaccine candidates are regulated. The results presented by Vembar et al. [9] are thus relevant for both drug development and vaccine design and provide ample opportunity for further investigation.

## Abbreviations

CITH: CAR-I and trailer hitch homolog
DOZI: Development of zygote inhibited
PfAlba: *P. falciparum* acetylation lowers binding affinity
